# The Improvement Effects of Intercropping Systems on Saline-Alkali Soils and Their Impact on Microbial Communities

**DOI:** 10.3390/microorganisms13071436

**Published:** 2025-06-20

**Authors:** Yan-Jun Wang, Gao-Xiang Qi, Na-Na Wang, Hong-Yun Dong, Yan Zhang, Han Lu, Ying Li, Hong-Cheng Wang, Xin-Hua Li, Hong-Yuan Liu

**Affiliations:** 1 State Key Laboratory of Nutrient Use and Management, Shandong Academy of Agricultural Sciences, Jinan 250100, China; wang.yanjun1985@163.com (Y.-J.W.); vip_qigx@163.com (G.-X.Q.); wangnana269@126.com (N.-N.W.); donghy0102@163.com (H.-Y.D.); swallow928@126.com (Y.Z.); lhan9408@163.com (H.L.); liyingsysu@189.cn (Y.L.); 13678823197@163.com (H.-C.W.); 2 National Technological Innovation Center for Comprehensive Utilization of Saline-Alkali Land, Dongying 257347, China

**Keywords:** intercropping, chemical properties, microbial community, soil organic matter, microbial networks

## Abstract

Saline-alkali soil has poor fertility and low organic matter content, which are key factors that limit agricultural productivity. Intercropping systems can enhance biodiversity in farmlands, thereby increasing the organic matter content. During this process, soil microorganisms respond to environmental changes. Therefore, we conducted a three-year intercropping enhancement experiment using saline-alkali soil. To avoid nutrient and microbial differences caused by the varying nutrient demands of different crop types, we systematically sampled the tillage layer of the soil (0–20 cm) from the subsequent crop (wheat season) in the intercropping systems. We found that compared to the control group, the three intercropping systems significantly increased the nutrient content in saline-alkali soil, including total nitrogen, total phosphorus, total potassium, organic matter, available nitrogen, and available potassium. Notably, there were significant increases in total nitrogen, organic matter, and available potassium. The intercropping systems had varying effects on the alpha and beta diversities of soil bacteria and fungi. Specifically, the effect of intercropping on fungal alpha diversity was significantly greater than that on bacterial alpha diversity, whereas its effect on bacterial beta diversity was greater than that on fungal beta diversity. Additionally, intercropping influenced microbial community composition, increasing the abundance of *Acidobacteria* and *Gemmatimonadetes* and decreasing the abundance of *Actinobacteria*. It also increased the abundance of *Ascomycota* and *Mortierella* and decreased the abundance of *Basidiomycota*. Total nitrogen and soil organic matter were identified as the primary environmental factors that significantly affected bacterial community composition; however, they had no significant impact on fungal communities. Intercropping had different effects on the fungal and bacterial networks. It increased the stability and complexity of the bacterial network. However, although it improved the stability of the fungal network, intercropping reduced its complexity. In summary, intercropping with leguminous plants is an effective way to enhance soil nutrients, particularly organic matter, in saline-alkali soils. Simultaneously, intercropping affects the soil microbial community structure of subsequent crops; however, the responses of bacteria and fungi to intercropping are significantly different. The results of this study provide data support for improving saline-alkali land through planting systems.

## 1. Introduction

Soil salinization and alkalization have long been major threats to terrestrial ecosystems [1]. Approximately 1 billion ha of land worldwide is experiencing soil salinization [2]. The global soil area affected by salinization is approximately 960 million ha, whereas the total area of various types of saline-alkali land in China is approximately 36 million ha, accounting for 4.9% of the country’s usable land area. Coastal saline-alkali land is a major component, with the Yellow River Delta being a typical representative [3]. Its unique geographical location and climatic factors have resulted in saline-alkali soils accounting for over 50% of the total area [4]. By integrating regional environmental characteristics, the establishment of a diversified and comprehensive management and utilization model for saline-alkali land in the Yellow River Delta could expand China’s arable land reserves [5,6]. This approach is important to maintain basic food self-sufficiency and ensure national food security.

Currently, the fertility of saline-alkali soils in the Yellow River Delta is poor, with extremely low organic matter content [7]. The local conventional planting system primarily involves wheat-maize (W-M) rotation, and long-term continuous planting has further exacerbated the decrease in soil nutrients. In recent years, intercropping systems have been increasingly applied to improve and utilize saline-alkali land owing to their high economic benefits and low environmental risks [8,9,10]. Compared with traditional monocultures, intercropping can balance resource utilization, enhance soil fertility, and reduce the risk of pest and weed outbreaks [11,12]. Certainly, intercropping has its drawbacks. For instance, competition for resources such as light, water, and nutrients may emerge among different crops, thereby necessitating rational crop pairing. Research shows that enhancing soil organic matter in intercropping systems increases soil permeability, which, in turn, elevates the leaching of soluble salt ions. Therefore, intercropping can reduce salt accumulation in the surface soil and redistribute salts throughout the entire soil profile, thereby alleviating the harmful effects of alkalization and secondary salinization on system productivity [10,12]. Legume-based intercropping can increase the biodiversity of soil ecosystems and is used worldwide [13,14]. Crop yield and soil fertility are enhanced through interspecies interactions, whereas legumes can improve nitrogen use efficiency in intercropping systems through nitrogen fixation and other mechanisms [15].

Coastal saline-alkali land is characterized by high salinity, severe leaching of soil humus, very low organic matter content, and damaged soil structure, primarily manifesting as soil compaction [16]. The soil is sticky when wet and hard when dry, and white salt deposits are often visible on the surface. Poor ventilation and water permeability severely affect crop growth, particularly during spring, when the returning salinity leads to low germination rates and reduced crop yields [17]. Therefore, one of our primary research objectives was to enhance the nutrient content of saline-alkali land, particularly by increasing the organic matter content and addressing soil compaction issues. The experimental site in this study has long been affected by land salinization and monoculture practices, resulting in nutrient depletion. Soil nutrient indicators, which are crucial for regional sustainable agricultural development, fall far short of these requirements. This study adopted a green ecological approach by selecting various functional plants suitable for cultivation in saline-alkali soil and pairing them with functional plant ecological control strips to enhance the self-regulation capacity of the soil. Additionally, based on current land conditions, we selected suitable crops for planting and designed intercropping systems to rationally improve the soil nutrient content and mitigate the compaction of saline-alkali soil [18].

Soil microorganisms play a crucial role in terrestrial ecosystems, and the formation and transformation of soil organic matter cannot occur without their involvement [19]. Microbial communities are sensitive to soil disturbances and changes in the external environment, and their diversity reflects the state of the soil ecological environment. Research has shown that soil microorganisms can restore the fertility of degraded saline-alkali land by fixing nitrogen and mobilizing key nutrients such as phosphorus and potassium to crop plants, thereby improving nutrient utilization efficiency and enhancing soil structure [20]. The structure of soil microbial communities is an important indicator of soil quality. The abundance and diversity of these communities are closely related to environmental factors such as farming practices and soil physicochemical properties.

This study presents experiments conducted on the saline-alkali land of the Yellow River Delta in Dongying, China, focusing on the effects of different planting systems on soil improvement (from 2020 onwards). We systematically analyzed the differences in the chemical properties of saline-alkali soil and the responses of microbial communities under four planting systems, including traditional wheat-maize rotation. The number of relevant studies on this topic is limited. To elucidate the influence of intercropping on microbial community structure, this study used soil samples collected during the harvest season of the following wheat crop to assess the effects of three intercropping planting modes on the nutrient content of saline-alkali soil and changes in microbial communities.

## 2. Materials and Methods

### 2.1. Field Experiment Site

This study presents experiments conducted in the saline-alkali lands of the Yellow River Delta National saline-alkali and Biological Agricultural Experimental Demonstration Park (37°17′–37°18′ N, 118°35′–118°37′ E), situated in Dongying City, Shandong Province, China, comprising coastal saline-alkali soils characterized by diverse crop types and cropping systems. The region belongs to the warm-temperate semi-humid continental monsoon climate zone, with an average annual temperature of 13.7 °C, annual precipitation of 582.6 mm, and annual evaporation > 1800 mm over the past 10 years. The annual temperature, precipitation, and humidity conditions are shown in Figure S1. The soil type is coastal saline-alkali, with a salinity of approximately 2–3‰. The experiment started in May 2021 and ended in June 2024, with a duration of 3 years.

### 2.2. Experimental Design

**Figure 1:**
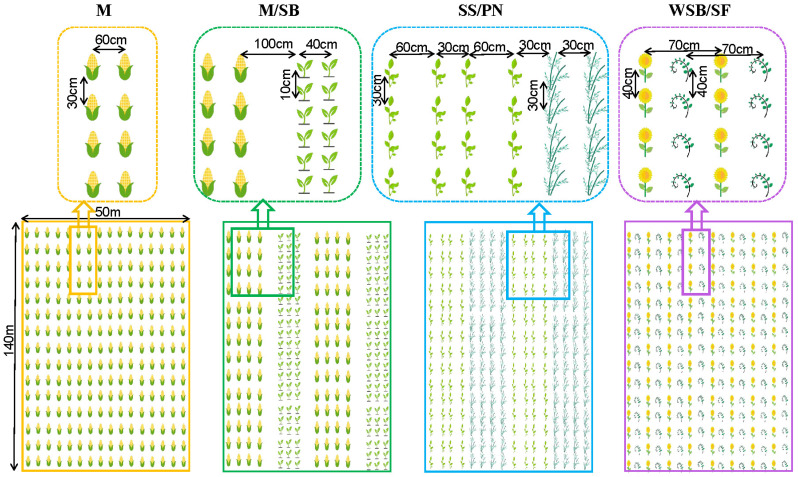
Diagram of field cropping system in the field. W-M, the wheat-maize monoculture system; W-M/SB, the wheat-maize/soybean intercropping system; W-SS/PN, the wheat-sesame/peanut intercropping system; W-WSB/SF, the wheat-wild soybean/sunflower intercropping system.

A randomized block design was used at the experimental site. During summer, maize (*Zea mays* L.)/soybeans (*Glycine max* L.) (M/SB), sesame (*Sesamum indicum* L.)/peanuts (*Arachis hypogaea* inn.) (SS/PN), and wild soybeans (*Glycine soja Sieb*. *Et Zucc*.)/sunflowers (*Helianthus annuus* L.) (WSB/SF) were established along with a maize (M) monoculture control group (Figure 1). During winter, wheat was planted in all plots. Each system consisted of three replicates, with each replicate representing a plot measuring 150 × 40 m, and a 10 m isolation strip was established between the plots. For the same intercropping system, the positions of the two intercropped plants were exchanged in the second year, and this was continued over three consecutive years.

In the maize monoculture system (W-M), the row spacing for maize was 60 cm with a plant spacing of 30 cm.

In the maize and soybean intercropping system (W-M/SB), both maize and soybeans were planted with equal row spacing, with the arrangement consisting of four rows of maize followed by three rows of soybeans. The row spacing for maize was 60 cm with a plant spacing of 30 cm, whereas the row spacing for soybeans was 40 cm with a plant spacing of 10 cm. The row spacing between the maize and soybeans was 100 cm.

In the sesame and peanut intercropping system (W-SS/PN), peanuts were planted in a wide–narrow row system, whereas sesame was planted with equal row spacing. The intercropping arrangement consisted of four rows of peanuts followed by four rows of sesame. The row spacing for sesame was 40 cm, with a plant spacing of 15 cm; the narrow row spacing for peanuts was 30 cm; and the wide row spacing was 60 cm, with a plant spacing of 30 cm. The row spacing between sesame and peanuts was 30 cm.

In the sunflower and wild soybean intercropping system (W-WSB/SF), the row spacing for sunflowers was 70 cm and the plant spacing was 40 cm, whereas the row spacing for wild soybeans was 70 cm and the plant spacing was 40 cm. Sunflowers are annual herbaceous plants with tall and robust stems, whereas wild soybeans are annual herbaceous plants. Therefore, an alternating planting system was adopted in which wild soybeans were planted between two rows of sunflowers, allowing them to climb and grow around the sunflower stems.

Winter wheat was planted with equal row spacing of 15 cm. The wheat seeding density was 300 kg/ha.

“Ludan9088” maize, “Huayu25” peanuts, “Qihuang34” soybeans, “Luzhi1” sesame, sunflowers (purchased from China Jiangsu Qixiu Seed Industry Co.), and wild soybeans (collected from China’s Dongying saline-alkali land) were sown in early June and harvested in early October. “Jimai60” wheat was sown in early October and harvested in early June. The base fertilizer for the wheat season was applied at 750 kg/hm^2^ using Jinzhengda compound fertilizer with an N:P_2_O_5_:K_2_O ratio of 15:15:15. Additionally, urea was applied during spring at a rate of 225 kg/hm^2^. During the maize season, seed fertilizer was applied alongside compound fertilizer at a rate of 600 kg/hm^2^ for all crops.

### 2.3. Soil Sampling and Soil Chemical Property Analysis

The soil samples for this study were collected before the wheat harvest in June 2024. Five soil samples were randomly collected from a depth of 0 to 20 cm and combined into a composite sample. Each plot contained three composite samples. These composite samples were placed in sterile plastic bags and transported to the laboratory at ultralow temperatures. Upon arrival at the lab, the samples were divided into two subsamples: the first subsample was stored at −80 °C for soil microbial diversity analysis, whereas the second subsample was used for soil chemical property analysis.

Methods for determining soil chemical properties: Soil pH was measured using a water–soil ratio of 1:2.5 (*w*/*v*). Soil organic matter (SOM) content was analyzed using the potassium dichromate oxidation method [21]. TN was analyzed using the Kjeldahl method, and available nitrogen (AN) was determined by calcium chloride extraction and measured using a flow analyzer [22]. Total potassium (TK) was analyzed using the hydrofluoric acid digestion method and flame photometry, whereas available potassium (AK) was analyzed using ammonium acetate extraction and flame photometry [23]. Available phosphorus (AP) was analyzed using the molybdenum–antimony anti-colorimetric method, and total phosphorus (TP) was determined by first melting the sample with sodium hydroxide and then analyzing it using the molybdenum–antimony anti-colorimetric method [24].

### 2.4. Analysis Methods for Soil Microbial Community Structure

The sequencing work for soil microbial diversity was performed by a professional sequencing company (Beijing Qinke Biotechnology Co., Ltd., Beijing, China). Bacterial and fungal DNA were extracted from fresh soil (0.5 g) using the CTAB/SDS method. The primers 338F/806R [25] and ITS1/ITS1R [26] with variable barcode sequences were used to amplify the bacterial 16S rRNA V3-V4 region and the fungal ITS1 region, respectively. PCR products were mixed in equal density ratios and purified using a gel extraction kit (Qiagen, Hilden, Germany). After the total DNA was extracted from the samples, PCR amplification was carried out. In order to determine the accuracy of DNA extraction and amplification, positive controls (standard samples containing bacteria and fungi) and negative controls (blank samples) were set up, respectively, during the processes of DNA extraction and amplification. Subsequently, the products were purified, quantified, and normalized to construct sequencing libraries. The constructed libraries were quality-controlled, and those that passed were sequenced using an Illumina NovaSeq 6000 (manufactured by Illumina, Inc., San Diego, CA, USA). Raw reads obtained from sequencing were filtered using Trimmomatic v0.33 software. Next, the primer sequences were identified and removed using cutadapt 1.9.1 software to obtain clean reads that did not contain primer sequences [27]. For the clustering method ‘denoising (dada2)’, we conducted further quality control, merged the paired-end reads, and removed chimeras using the dada2 package in R. For the clustering method ‘similarity clustering’, we utilized USEARCH (version 10) to merge paired-end reads and eliminate chimeras (UCHIME [4], version 8.1), ultimately yielding high-quality sequences for subsequent analysis. The DADA2 method in QIIME2 2020.6 was used for denoising, paired-end sequence merging, and the removal of chimeric sequences to obtain the final valid data (non-chimeric reads) [28]. The taxonomic database is Silva (Release 138, http://www.arb-silva.de, accessed on 20 January 2025).

### 2.5. Statistical Analysis

The data analysis of soil chemical properties was conducted using Excel software, and graphs were created using OriginPro 2024. The alpha diversity (Chao 1 richness and Shannon diversity) was calculated using QIIME2, and the number of reads was normalized. The beta diversity was evaluated using non-metric multidimensional scaling (NMDS) based on the Bray-Curtis distance, conducted with the *vegan* package. Based on the abundance of and variation in each species in the samples, Spearman rank correlation analysis (default method) was performed, and a correlation network was constructed by selecting data with a correlation greater than 0.1 and a *p*-value less than 0.05. We calculated the network topological properties to compare microbial associations and the complexity of networks across different treatments, including the number of nodes and edges, average degree, average path length, graph density, clustering coefficient, betweenness centralization, degree centralization, and modularity. Nodes in the network that represented module hubs, network hubs, and connectors were identified as keystone species [29](. The interactions between the microbial community and environmental factors were assessed using Sparse Correlations for Compositional data (SparCC) [30], the Mantel test, and redundancy analysis (RDA). Analysis and visualization were performed using the R-based *vegan* package (v2.3) and Gephi (0.10.1) [31].

## 3. Results

### 3.1. The Impact of Intercropping Systems on Soil Chemical Properties

**Figure 2:**
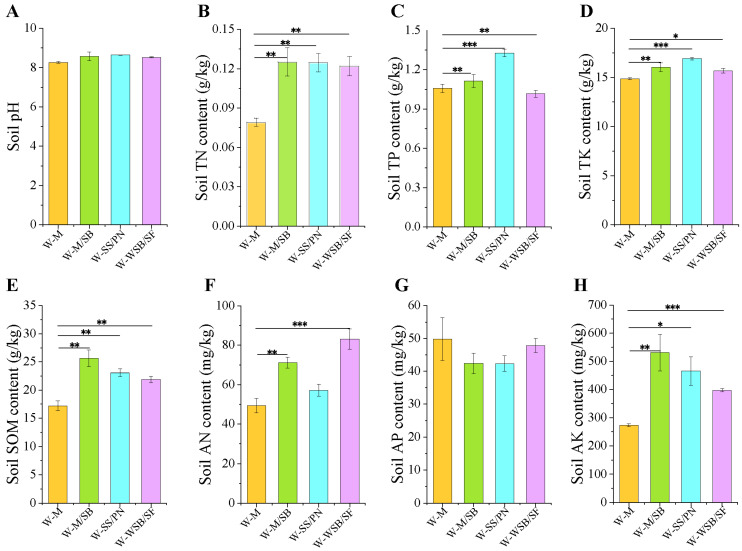
Soil chemical properties. (**A**) The soil pH. (**B**) The soil total nitrogen (TN) content. (**C**) The soil total phosphorus (TP) content. (**D**) The soil total potassium (TK) content. (**E**) The soil organic matter (SOM) content. (**F**) The soil available nitrogen (AN) content. (**G**) The soil available phosphorus (AP) content. (**H**) The soil available potassium (AK) content. * *p* < 0.05, ** *p* < 0.01, *** *p* < 0.001.

Under the same fertilization conditions, we analyzed soil chemical properties under the four cropping systems (Figure 2). Compared with the traditional maize monoculture (W-M), the intercropping systems did not significantly affect soil pH (Figure 2A) or AP (Figure 2G) but could effectively enhance soil TN (Figure 2B), TP (Figure 2C), TK (Figure 2D), SOM (Figure 2E), AN (Figure 2F), and AK (Figure 2H) content. The TN content increased by approximately 57% in all three intercropping systems compared with that in W-M (Figure 2B). The best TP and TK enhancement was achieved by W-SS/PN, with increases of 25.5% (Figure 2C) and 12.0% (Figure 2D), respectively, compared with W-M. Additionally, soil AN (Figure 2F), AK (Figure 2H), and SOM (Figure 2E) content significantly increased in the three intercropping planting systems. Compared to the control group, the AN content of W-M/SB, W-SS/PN, and W-WSB/SF increased by 44.2, 15.9, and 68.4%, respectively; the AK content increased by 94.1, 70.4, and 45.4%, respectively; and the SOM content increased by 49.1, 34.0, and 27.3%, respectively. The enhancement of nutrients directly promotes an increase in crop yields (Figure S2).

### 3.2. Diversity and Structure of Soil Bacterial and Fungal Communities

**Figure 3:**
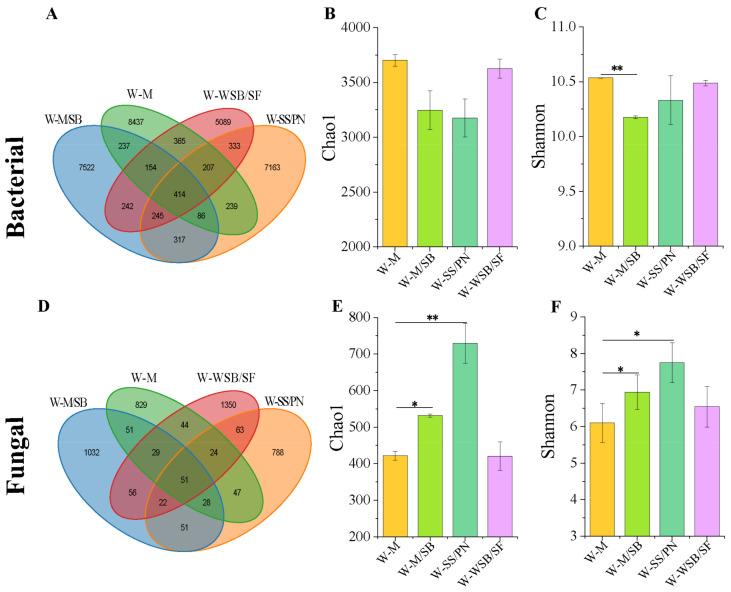
Venn diagram of fungi and bacteria and alpha diversity analysis. (**A**) The Venn diagram of bacteria. (**B**) The Chao1 index of bacteria. (**C**) The Shannon index of bacteria. (**D**) The Venn diagram of fungi. (**E**) The Chao1 index of fungi. (**F**) The Shannon index of fungi. * *p* < 0.05, ** *p* < 0.01.

A total of 886,452 clean bacterial community reads and 747,617 fungal community sequences were obtained by high-throughput sequencing of the 12 samples. The data were processed to yield a total of 31,050 bacterial and 4465 fungal amplicon sequence variant (ASV) classifications. The number of shared ASVs among the bacterial communities across the four planting systems was 414 (Figure 3A), whereas the number of shared ASVs among the fungal communities was 51 (Figure 3D). The number of unique bacterial ASVs in the three intercropping systems, W-M/SB, W-WSB/SF, and W-SS/PN, was lower than that in W-M. This indicates that the intercropping systems did not increase the number of unique bacterial ASVs in the soil. In contrast to the bacterial results, the number of unique fungal ASVs in the W-M/SB and W-WSB/SF intercropping systems was significantly higher than that in W-M. However, the number of unique fungal ASVs was lower in the W-SS/PN group than in the W-M group. These results suggest that intercropping systems can increase the number of unique ASVs in soil fungal communities; however, this effect is influenced by factors such as crop type.

The Chao1 (richness) and Shannon (diversity) indices were used for the alpha diversity analysis. The results showed that the Shannon index of W-M/SB was significantly lower than that of W-M (*p* < 0.05), whereas the Chao1 and Shannon indices of the other two groups of bacterial communities were slightly lower than those of W-M (*p* > 0.05) (Figure 3B,C). The changes in the fungal Chao1 and Shannon indices were quite different from those of the bacteria. The Chao1 and Shannon indices of the fungi were higher than those of W-M in all three intercropping systems (Figure 3E,F). The alpha diversity indices of the two intercropping systems W-M/SB and W-SS/PN were significantly higher than those of W-M (*p* > 0.05). These results indicate that intercropping had no significant effect on bacterial richness and diversity but could significantly enhance the richness and diversity of fungal communities.

**Figure 4:**
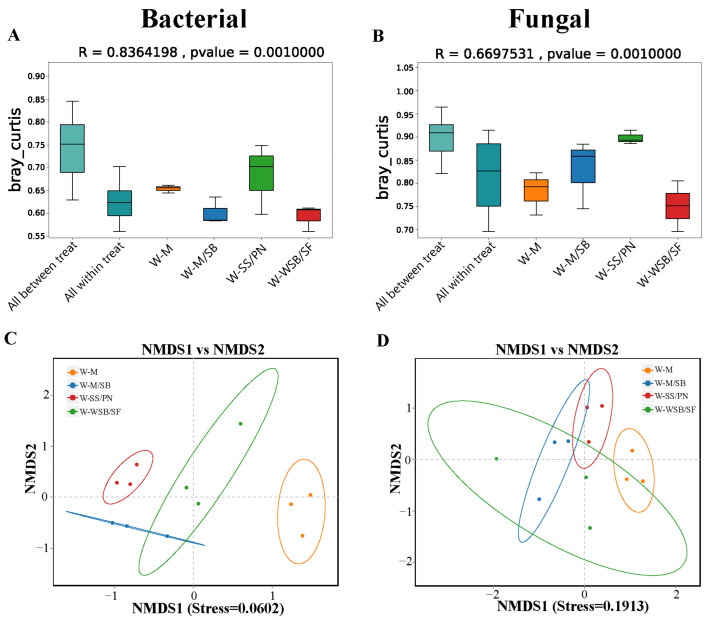
Beta diversity analysis. Boxplot of PERMANOVA/ANOSIM analysis for bacteria (**A**) and fungi (**B**). Non-metric multidimensional scaling (NMDS) analysis plot of bacteria (**C**) and fungi (**D**).

The analysis of similarities based on the distance matrix between the samples showed that the R-values for the bacterial and fungal communities were 0.8364 and 0.6698, respectively, with *p* = 0.001 (Figure 4A,B). This indicates that the differences between the bacterial and fungal communities in the four cropping systems were statistically significant. An R-value closer to one suggests that the samples are more similar within a group and more distinct from those of other groups. Additionally, a *p*-value of <0.05 confirms the significance of the differences between the groups. The NMDS ordination of the community data based on the Bray-Curtis distance showed that soil bacterial (Figure 4C) and fungal (Figure 4D) communities differed significantly among the four cropping systems. Intercropping had a greater effect on the bacterial community structure than on the fungal community structure. The stress coefficients for the bacterial and fungal communities were 0.0602 (<0.1) and 0.1913 (<0.2), respectively, further validating these findings (Figure 4C,D).

### 3.3. Soil Bacterial and Fungal Community Compositions

**Figure 5:**
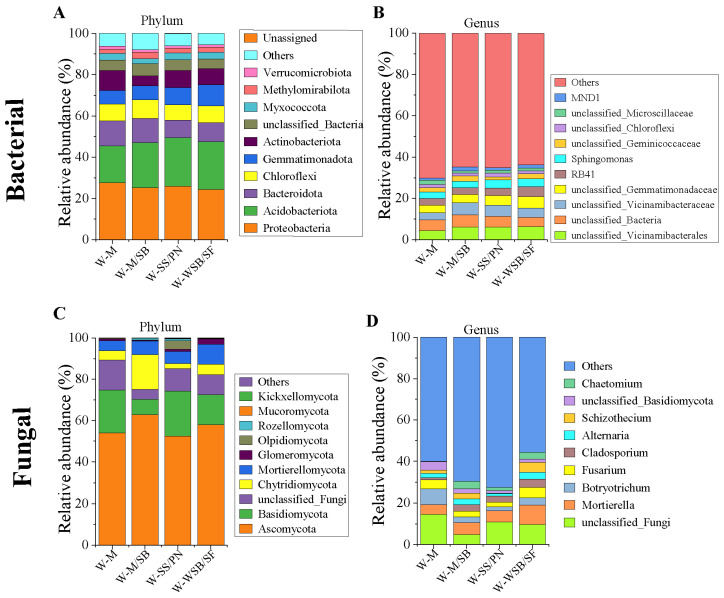
Species distribution analysis. Relative abundance of dominant phyla (>1%) in bacterial (**A**) and fungal (**C**) communities. Relative abundance of dominant genus (>1%) in bacterial (**B**) and fungal (**D**) communities.

We analyzed the abundance at the phylum and genus levels. The most abundant bacterial phyla were *Proteobacteria* (24.29–27.66%) *Acidobacteriota* (17.88–23.62%), *Bacteroidota* (8.57–12.07%), *Chloroflexi* (7.30–8.98%), *Gemmatimonadota* (6.66–10.25%), *Actinobacteriota* (4.81–9.65%), *Myxococcota* (2.29–3.39%), and *Methylomirabilota* (1.87–3.15%). All three intercropping systems increased the abundance of *Acidobacteriota*, *Gemmatimonadota*, and *Methylomirabilota*, but decreased the abundance of *Proteobacteria*, *Actinobacteriota*, and *Bacteroidota* (Figure 5A and Figure S3). Furthermore, genus-level analysis revealed that the abundance of dominant bacterial genera in the intercropped soils was significantly higher than that in the W-M soil (Figure 5B).

Fungi were primarily distributed among the following five phyla: *Ascomycota* (52.49–62.70%), *Basidiomycota* (7.62–21.73%), *Chytridiomycota* (2.42–16.89%), *Mortierellomycota* (4.80–9.72%), and *Glomeromycota* (0.35–2.70%) (Figure 5C). There were significant differences in the abundances of the dominant fungal phyla (Figure 5C and Figure S4) and genera (Figure 5D) among the four planting systems, indicating that the planting system had a much greater impact on fungal community structure than on bacterial communities. From the changes in the abundance of fungal phyla, it can be observed that the three intercropping systems have inconsistent effects on the dominant fungal groups, with W-M/SB having the greatest impact on fungal community structure. Compared to W-M, W-M/SB significantly increased the abundance of *Ascomycota* and *Chytridiomycota* and significantly decreased the abundance of *Basidiomycota* (Figure S4). Notably, all three intercropping systems increased the abundance of *Ascomycota* and *Mortierella* and decreased the abundance of *Basidiomycota*.

### 3.4. Differences in the Soil Bacterial and Fungal Networks Under Different Cropping Systems

**Figure 6:**
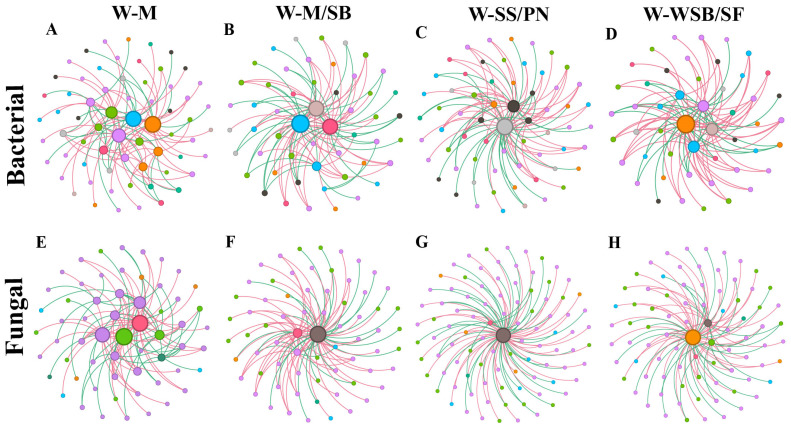
Correlation network analysis of bacteria (**A**–**D**) and fungi (**E**–**H**) at the genus level under different cropping systems. The modules in each network are indicated in different colors, and the node size in each network is proportional to the degree.

To investigate the effects of different cropping systems on the soil microbial community networks, we analyzed the bacterial and fungal community networks at the genus level for each cropping system (Figure 6, and Table 1). We selected 100 microbial correlation connections of high strength (edges_num) to construct the network, thereby highlighting the primary microbial interaction relationships. The overall topological analysis revealed that the bacterial and fungal network parameters in the soils of the four cropping systems exhibited some differences.

In both the bacterial and fungal networks, the node connectivity and edge connectivity of the four planting systems are 1. This implies that in terms of the connection stability of nodes and edges, the network stability of the four planting systems is relatively good. It is worth noting that compared with the W-M (monoculture) system, the average path lengths of bacteria and fungi in the intercropping systems are shorter. A shorter average path length can accelerate the transmission speed of interactions among microorganisms, enabling the network to respond more promptly to disturbances. This, to some extent, reflects that the network stability of the intercropping systems is better. In addition, compared with the W-M system, the graph diameter of the intercropping systems is smaller, indicating that the maximum distance of interactions among microorganisms in the intercropping systems is shorter and the network structure is more compact. When comparing the intercropping systems and the monoculture system, there are differences in the number of nodes. Some intercropping systems have more nodes, which may increase the microbial species richness and thus enhance the network complexity. In the bacterial network, the average degree and graph density of the intercropping systems are mostly higher than those of the monoculture system, and there are significant differences in some clustering coefficients. This makes the interactions among bacteria more frequent and intensive, changes the local aggregation structure, and makes the network more complex. However, in the fungal network, the average degree, graph density, and clustering coefficients of the intercropping systems are all lower than those of the monoculture system, and the complexity of their interactions is decreased. In addition, the modularity values of the intercropping systems vary, which may change the network module structure and affect the network complexity.

In summary, compared with monoculture, the three intercropping methods enhance the stability of the bacterial and fungal networks. On this basis, intercropping increases the complexity of the bacterial network but reduces the complexity of the fungal network.

The keystone species in the bacterial networks primarily belonged to *Acidobacteriota*, *Proteobacteria*, *Bacteroidota*, and *Gemmatimonadota*. In the fungal networks, the keystone species belonged to *Ascomycota*, *Mortierellomycota*, and *Basidiomycota* (Figure 6, and Table 2). In both the bacterial and fungal networks of the four cropping systems, each network contains 2–3 module hubs (Zi > 2.5 and Pi < 0.62). However, the numbers of network hubs (Zi > 2.5 and Pi > 0.62) and connectors (Zi < 2.5 and Pi > 0.62) vary greatly. Notably, in the bacterial network, W-M/SB has neither network hubs nor connectors. In the fungal network, W-M, W-M/SB, and W-SS/PN have no network hubs. Specifically, W-SS/PN has neither network hubs nor connectors. Overall, there are certain differences in the bacterial and fungal networks of the four cropping patterns. However, in this study, no common patterns induced by the monocropping or intercropping planting methods were found.

### 3.5. The Relationship Between Microbial Communities and Soil Chemical Properties

**Figure 7:**
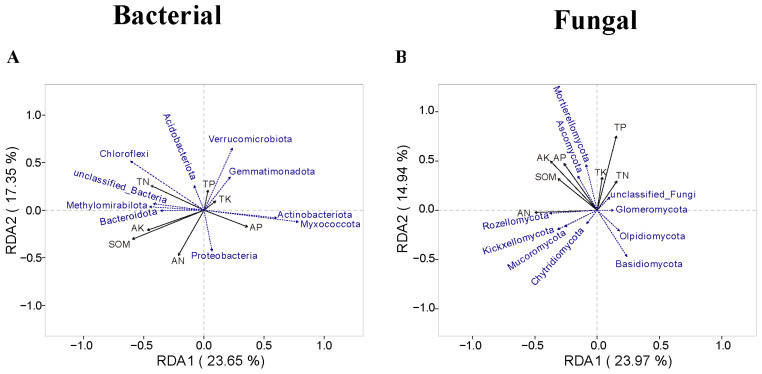
The relationship between dominant bacterial (**A**) and fungal (**B**) genera and soil chemical properties. The blue arrows and labels represent microbial taxa, and the black arrows and labels represent soil properties.

**Figure 8:**
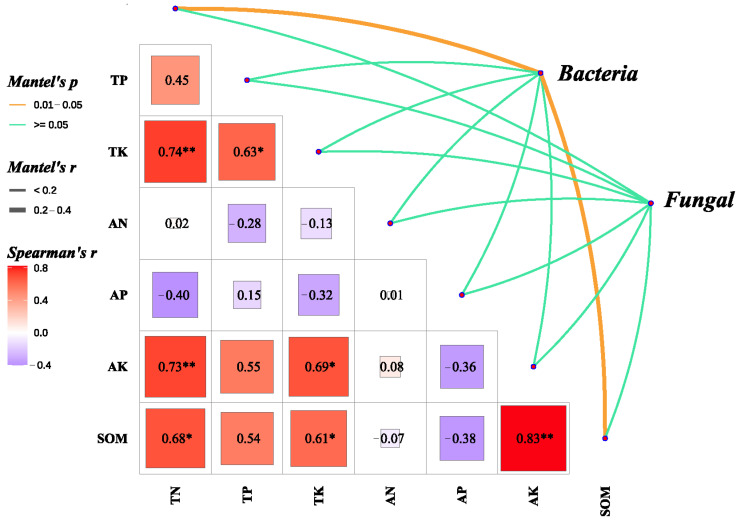
Spearman’s correlation between the relative abundance of bacteria and fungi at the phylum level and environmental factors. TN, total nitrogen content. TP, total phosphorus content. TK, total potassium content. SOM, soil organic matter content. AN, available nitrogen content. AP, available phosphorus content. AK, available potassium content. * *p* < 0.05, ** *p* < 0.01.

A redundancy analysis (RDA) of soil microbial communities and chemical properties was performed at the genus level. The results showed that the bacterial communities *Acidobacteriota*, *Chloroflexi*, *Methylomirabilota*, and *Bacteroidota* were positively correlated with TN, AK, SOM, and AN. In contrast, *Proteobacteria*, *Myxococcota*, *Actinobacteriota*, *Gemmatimonadota*, and *Verrucomicrobiota* were positively correlated with TP, TK, and AP (Figure 7A). *Ascomycota* and *Mortierellomycota* in the fungal community were positively correlated with TP, TN, TK, AK, AP, and SOM; however, these correlations were not significant, with 14.94% of the variance explained by RDA2 (Figure 7B). We further analyzed the effect of soil chemical properties on both fungal and bacterial community structures and found that TN and SOM had a significant effect on the bacterial community structure (Figure 8). However, the effect of soil chemical properties on the fungal community was not significant compared to that on the bacterial community, and we did not identify any environmental factors that significantly affected the fungal community.

Combined with the analysis of community structure and diversity, the factors influencing the fungal community were more complex than those affecting the bacterial community, with the latter being relatively more stable.

## 4. Discussion

### 4.1. Intercropping System Can Effectively Enhance Soil Nutrient Content in Saline-Alkali Soil

The three intercropping systems had no significant effects on soil pH compared to that of W-M (Figure 2A), indicating that intercropping systems do not significantly reduce soil alkalinity. In this study, three legumes-soybeans, peanuts, and wild soybeans–were intercropped to enhance the soil nitrogen content through their nitrogen-fixing capabilities. The results met these expectations, as TN and AN content were significantly enhanced in all three intercropping systems (Figure 2B,F). These findings were consistent with those of previous studies [13,14,15]. Additionally, AN content in the W-SS/PN intercropping system was significantly lower than that in the W-M/SB and W-WSB/SF systems. However, TP (Figure 2C) and TK (Figure 2D) content were significantly higher in the W-SS/PN system than in the other two groups. These differences are likely due to variations in nitrogen fixation capacity and nutrient requirements among different crops. It is worth noting that the TP content in all three intercropping groups was significantly higher than that in W-M. However, the AP content was slightly lower in these groups than that in the W-M group, although none of these differences were statistically significant (Figure 2G). This indicates that intercropping can enhance TP content. However, factors such as the alkalinity of saline-alkali soils, clayey texture, and other conditions may reduce the effectiveness of phosphorus availability [31].

Compared with the monocultures, the intercropping systems of the three different crops significantly enhanced nutrient levels, particularly SOM (Figure 2E), AN (Figure 2F), and AK (Figure 2H), which showed extremely significant increases. Except for AP, all other soil nutrient indices reached levels comparable to those of non-saline-alkali soils. Notably, the SOM increased annually throughout the three-year planting experiments, clearly demonstrating that intercropping can effectively enhance SOM content in saline-alkali soils [32].

### 4.2. Intercropping System Effects on Microbial Community Structures

In this study, the results of the alpha diversity index indicated that the intercropping system had a slight effect on the richness and abundance of bacteria (Figure 3B,C), albeit a more pronounced effect on their community structure (Figure 4A,C). However, intercropping had the opposite effect on fungi, with combinations such as W-M/SB and W-SS/PN significantly enhancing fungal community richness and abundance (Figure 3E,F). This result contrasts with findings from previous studies, which reported that bacteria tended to respond faster to intercropping treatments than fungi [33,34]. This may be because this study was conducted in saline-alkali soils, where fungi are less tolerant to salinity and more sensitive to environmental stresses than bacteria, and therefore respond more strongly to the environment. Intercropping with W-WSB/SF had no significant effect on the richness and abundance of either bacteria or fungi (Figure 3). This suggests that intercropping was not the primary factor affecting microbial richness and abundance. As confirmed in previous studies [35], other environmental factors, such as crop species, nitrogen content, and soil nutrients, such as organic matter, may have a greater influence. Additionally, bacteria are more affected by soil chemical properties, whereas fungi are more dependent on vegetation [36,37,38].

The dominant bacterial phyla reported to have a relatively high abundance at the phylum level in saline-alkali soils include *Proteobacteria*, *Actinobacteriota*, *Bacteroidota*, *Chloroflexi*, *Acidobacteriota*, *Gemmatimonadota*, and *Verrucomicrobia*. Fungi with higher abundance at the phylum level include *Ascomycota*, *Basidiomycota*, *Chytridiomycota*, *Mortierellomycota*, and *Glomeromycota* [39,40,41]. Most of the results obtained in this study are consistent with these findings; however, there were significant differences in the abundances of some dominant phyla, such as a lower abundance of *Actinobacteriota* and a higher abundance of *Acidobacteriota* and *Gemmatimonadota*. Intercropping increased the abundance of *Acidobacteriota* and *Gemmatimonadota*, which are not typically characterized as fast-growing eutrophic bacteria, but decreased the abundance of *Actinobacteriota*, which are slow-growing oligotrophic bacteria. *Acidobacteriota* are widely distributed in the soil and are often abundant in acidic and less fertile soils. They are an important component of the soil microbial community and play crucial roles in processes such as soil formation, nutrient cycling, and plant growth. Despite this, *Acidobacteriota* typically prefer acidic soils, and because saline-alkali soils are alkaline, their abundance is relatively low. Additionally, intercropping significantly increased the abundance of the *Ascomycota* and *Mortierella* fungal communities. According to fungal network analysis, these fungi are key indicator species in fungal networks. They are widely distributed in soil, plant residues, bark, and leaf litter and are primarily involved in organic matter degradation [36,37,38]. The abundance of these fungi was correlated with SOM, which was consistent with the soil chemical results. Among the four cropping systems, *Botryotrichum* abundance was highest in the W-M system. This indicates that intercropping systems can reduce pathogenic microbial abundance, increase probiotic flora abundance, and optimize the structure of soil flora compared to the W-M system [42,43]. This further suggests that intercropping enhances soil nutrient content, particularly SOM, and that the dominant microbial populations change as the proportion of readily decomposable and refractory organic matter changes. The structure of the soil microbial community is closely related to soil nutrient content [44].

### 4.3. Intercropping System Effects on Microbial Community Network

Microorganisms engage in intricate interactions, which give rise to specific microbial networks. These networks are crucial for maintaining and stabilizing the functions of the soil ecosystem. Currently, a multitude of methods exist for studying microbial networks, and as reported in *ISME* [45], different analytical approaches can yield significantly divergent results. To ensure the accuracy of the analysis in this study, we constructed networks exclusively for the dominant microbial communities with high abundances. We adopted this approach to minimize the likelihood of false positives, thereby enhancing the reliability of our findings. Several studies have proposed factors influencing the complexity of microbial networks, such as precipitation [44], salt stress, carbon dioxide concentration [33], and global warming [46], among others. The results of the current study indicate that intercropping has different effects on bacterial and fungal networks. This difference mainly stems from the disparities in the inherent characteristics of microorganisms, the environmental conditions created by intercropping, and the distinct functional roles of the two in the ecosystem. Additionally, differences in crop type, biomass, and soil nutrients may lead to different responses in fungi [37,38]. The intercropping effects on soil microbial networks vary under different habitat conditions. Several studies have shown that intercropping can increase the complexity of microbial network structures during the current season, whereas a few studies have suggested that intercropping has only a minor effect on microbial networks. For example, Peng et al. [44] confirmed that drought can significantly reduce microbial network complexity. However, under equivalent rainfall conditions, intercropping can also increase microbial network complexity. Additionally, studies [47] have indicated that in nitrogen-fertilized soybean/corn and peanut/corn systems, intercropping increased the complexity and stability of bacterial networks. Conversely, in the absence of nitrogen, the complexity and stability of the bacterial networks in a soybean–maize intercropping system were reduced. Compared with abiotic stress factors, the influence of intercropping on the network complexity of the topsoil was relatively weak, which is also supported by the results of this study.

Most research reports indicate that intercropping with leguminous plants can enhance the diversity of arbuscular mycorrhizal fungi and alter their community composition [47]. The sampled habitats in this study differed significantly from those reported in previous studies. Under the habitat conditions of this study, the three intercropping patterns (W-M/SB, W-SS/PN, and W-WSB/SF) reduced the complexity of the fungal network. This reduction also reveals that the response mechanisms of fungi and bacteria to the environment vary considerably. These findings offer a valuable addition to existing research.

In addition, compared with the monocropping system W-M, the key indicator species in the intercropping systems have changed in terms of phyla and quantity. The impacts of different intercropping systems on the key indicator species in bacterial and fungal networks vary, indicating that intercropping can alter the soil microbial community structure, but the specific impacts depend on the types of crops and intercropping methodologies.

### 4.4. Importance of Enhancing SOM in Saline-Alkali Soils

The organic matter content of saline-alkali soils is typically low, in the range of 1–2% or even lower [48]. The experimental site of this study is a coastal saline-alkali land in the Yellow River Delta, dominated by soluble salts (predominantly NaCl) with a soil pH ranging from 8.2 to 8.8. The surface soil layer (0–20 cm) exhibits high salt content, while the deep soil has relatively lower salinity. However, influenced by groundwater, soil salinity varies significantly with seasonal fluctuations, featuring severe salt accumulation in spring that consequently results in low seedling emergence rates during this period. The high salinity of these soils inhibits the growth of most crops, resulting in low seedling emergence and biomass. This significantly reduces the amount of organic residue added to the soil, thus affecting SOM accumulation [16]. Additionally, a high-salt environment adversely affects the survival and reproduction of soil microorganisms, thereby reducing their activity. Under salt stress, plants adapt to saline environments through osmotic adjustment, ion transport, antioxidant systems, and hormonal signaling, while soil microbes synergistically enhance plant salt tolerance by promoting osmolyte synthesis and optimizing ion homeostasis. Thus, the composition of soil microbial communities is crucial for crop salt stress resistance. Microorganisms play key roles in promoting organic matter transformation and accumulation [36,49]. However, reduced microbial activity slows down the decomposition of organic matter, making it challenging to achieve effective organic matter accumulation. In this study, an intercropping rotation planting system was implemented over three years. The results showed that the SOM content of the three intercropping systems exceeded 2%. Specifically, the organic matter content of the W-M/SB intercropping system reached 2.5%, representing a 49% increase compared with that of W-M (Figure 2E). The findings of this study demonstrate that intercropping with legumes is an effective strategy for enhancing the organic matter content of saline-alkali soils compared with conventional maize monocropping. Additionally, the results of the Mantel test analysis showed that TN and SOM significantly affected the bacterial community structure (Figure 7) but had no significant effect on the fungal community. This finding differs from those reported by Ma et al. [35]. This may be because as plant litter decomposes, the composition of the decomposition products changes, leading to differences in the microbial community structure. Consequently, intercropping resulted in variations in the soil microbial community structure during the current and subsequent seasons. Microbial communities respond differently to various habitats, and their responses to intercropping vary across soil environments [35,36].

In this study, the intercropping systems discussed still present numerous research gaps regarding soil organic matter (SOM) improvement. The efficiency of the three intercropping methods in enhancing SOM was not directly proportional to the biomass of straw returned to the field. Furthermore, aboveground litter biomass in the intercropping systems was not systematically quantified, and the molecular mechanisms underlying microbe–plant interactions under intercropping conditions remain undefined. These questions will be addressed through in-depth investigations in our future studies.

## 5. Conclusions

Intercropping is an effective method for enhancing SOM in saline-alkali soils. This nutrient enhancement effect is influenced by the crop species used. Although intercropping did not significantly affect the alpha diversity of soil bacteria in the next crop season, it influenced the abundance of the dominant bacterial community and altered its beta diversity. Intercropping significantly affected the alpha diversity of soil fungal communities during the next crop season. However, its effect on the beta diversity of the fungal communities was lower than that on the bacterial communities. The long-term effects of intercropping on the complexity and stability of the fungal and bacterial networks in the crops during the next season were weak. The bacterial community structure responded significantly to the TN and SOM content, whereas fungi did not respond significantly to the soil chemical properties. In this study, we confirmed the effects of three intercropping systems, maize/soybeans, sesame/peanuts, and wild soybeans/sunflowers, on nutrient enhancement in saline-alkali soils. We also analyzed the characteristics of soil microbial responses during the next growing season in these intercropping systems. The results provide a theoretical basis for the improvement and practical use of saline-alkali soils.

## Figures and Tables

**Table 1:** Topological properties of empirical networks of bacteria and fungi between different cropping systems.

Network Indexes	Bacterial				Fungal			
	W-M	W-M/SB	W-SS/PN	W-WSB/SF	W-M	W-M/SB	W-SS/PN	W-WSB/SF
nodes_num	65	52	63	49	59	68	94	82
edges_num	100	100	100	100	100	100	100	100
average_degree	3.077	3.846	3.175	4.082	3.390	2.941	2.128	2.439
nodes_connectivity	1	1	1	1	1	1	1	1
edges_connectivity	1	1	1	1	1	1	1	1
average_path_length	3.588	2.523	2.424	2.300	2.961	2.059	1.996	2.557
graph_diameter	36	36	24	24	24	18	18	24
graph_density	0.048	0.075	0.051	0.085	0.058	0.044	0.023	0.030
clustering_coefficient	0.058	0.005	0.027	0.086	0.052	0.039	0.005	0.004
betweenness_centralization	0.503	0.512	0.724	0.523	0.381	0.868	0.994	0.873
degree_centralization	0.264	0.552	0.691	0.644	0.286	0.867	0.966	0.748
modularity	0.529	0.295	0.360	0.247	0.439	0.280	0.131	0.400

**Table 2:** Taxa of keystone species of bacteria and fungi between different cropping systems.

	Module Hubs		Network Hubs		Connectors	
	Num.	Plylum	Num.		Num.	
**Bacterial Taxa**						
W-M	3	Acidobacteriota; Chloroflexi; Bacteroidota	1	Proteobacteria	5	Proteobacteria (2); Bacteroidota; Gemmatimonadota
W-M/SB	3	Acidobacteriota; Gemmatimonadota; unclassified bacteria	0		0	
W-SS/PN	2	Acidobacteriota; unclassified bacteria	2	Patescibacteria; Bacteroidota	0	
W-WSB/SF	2	Proteobacteria; unclassified bacteria	2	Acidobacteriota; Gemmatimonadota	9	Proteobacteria (4); Bacteroidota; Actinobacteriota (3); Myxococcota
**Fungal Taxa**						
W-M	3	Ascomycota; Basidiomycota; unclassified fungi	0		5	Ascomycota (5)
W-M/SB	3	Ascomycota; Mortierellomycota; unclassified fungi	0		3	Ascomycota; Mortierellomycota; unclassified fungi
W-SS/PN	2	Mortierellomycota; unclassified fungi	0		0	
W-WSB/SF	2	Mortierellomycota; unclassified fungi	1	Basidiomycota	2	Ascomycota; Basidiomycota

## Data Availability

The original contributions presented in this study are included in the article/Supplementary Material. Further inquiries can be directed to the corresponding authors.

## Supplementary Materials

The following supporting information can be downloaded at: https://www.mdpi.com/article/10.3390/microorganisms13071436/s1, Figure S1: Temperature (A), precipitation (B), and humidity (C) in the experimental field throughout the year (2024). Figure S2: The yield of wheat grains and straw. * *p* < 0.05, ** *p* < 0.01, *** *p* < 0.001. Figure S3: Species difference analysis of bacteria at the phylum level. (A) The relative abundance of Proteobacteria. (B) The relative abundance of Gemmatimonadota. (C) The relative abundance of Bacteroidota. (D) The relative abundance of Acidobacteriota. (E) The relative abundance of Actinobacteriota. (F) The relative abundance of Methylomirabilota. * *p* < 0.05, ** *p* < 0.01, *** *p* < 0.001. Figure S4: Species difference analysis of fungi at the phylum level. (A) The relative abundance of *Ascomycota*. (B) The relative abundance of *Mortierellomycota*. (C) The relative abundance of *Basidiomycota*. (D) The relative abundance of *Chytridiomycota*.* *p* < 0.05, ** *p* < 0.01, *** *p* < 0.001.

## Abbreviations

The following abbreviations are used in this manuscript:

M/SB.	Maize/soybeans
SS/PN	Sesame/peanuts
WSB/SF	Soybeans/sunflowers
M	Maize
W-M	The maize monoculture system
W-M/SB	The maize and soybean intercropping system
W-SS/PN	The sesame and peanut intercropping system
W-WSB/SF	The sunflower and wild soybean intercropping system
SOM	Soil organic matter
TN	Total nitrogen
AN	Available nitrogen
TP	Total phosphorus
AP	Available phosphorus
TK	Total potassium
AK	Available potassium
