# Supplementary material for: The Improvement Effects of Intercropping Systems on Saline-Alkali Soils and Their Impact on Microbial Communities
Source: Microorganisms. 2025 Jun 20;13(7):1436. doi: 10.3390/microorganisms13071436 (PMC12300658; doi:10.3390/microorganisms13071436)
Supplement: Supplementary file 1 [file microorganisms-13-01436-s001.zip › microorganisms-3636860-supplementary.pdf]

Figure S1

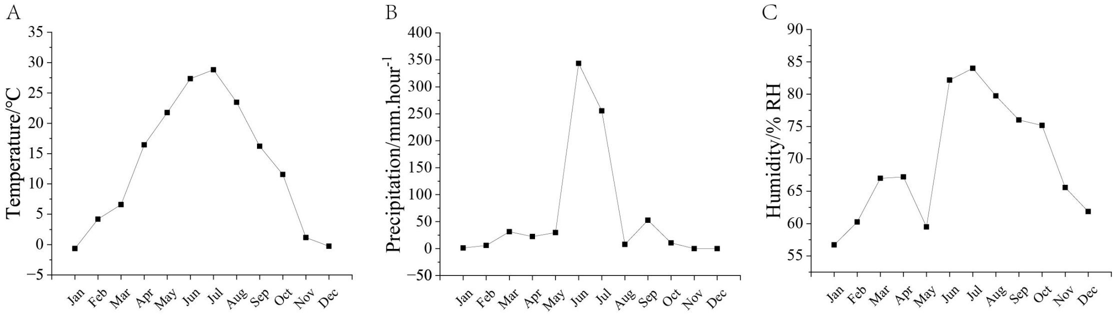

Figure S1Temperature(A), precipitation(B), and humidity(C) in the experimental field throughout the year. (2024)

Figure S2

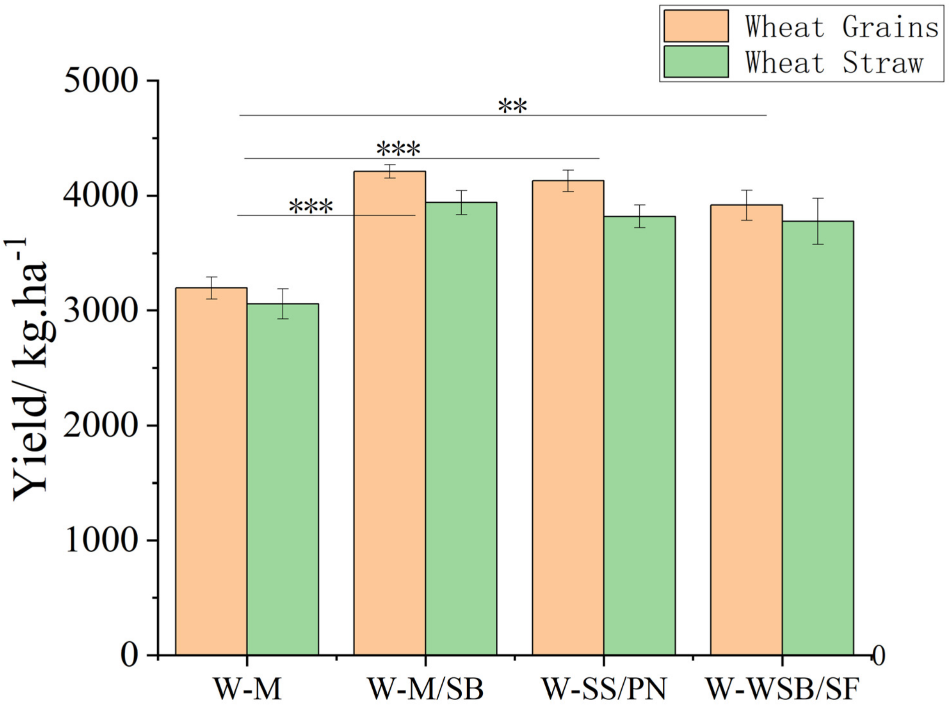

Figure S2 The yield of wheat grains and straw. * $p < 0.05$, ** $p < 0.01$, *** $p < 0.001$.

Figure S3

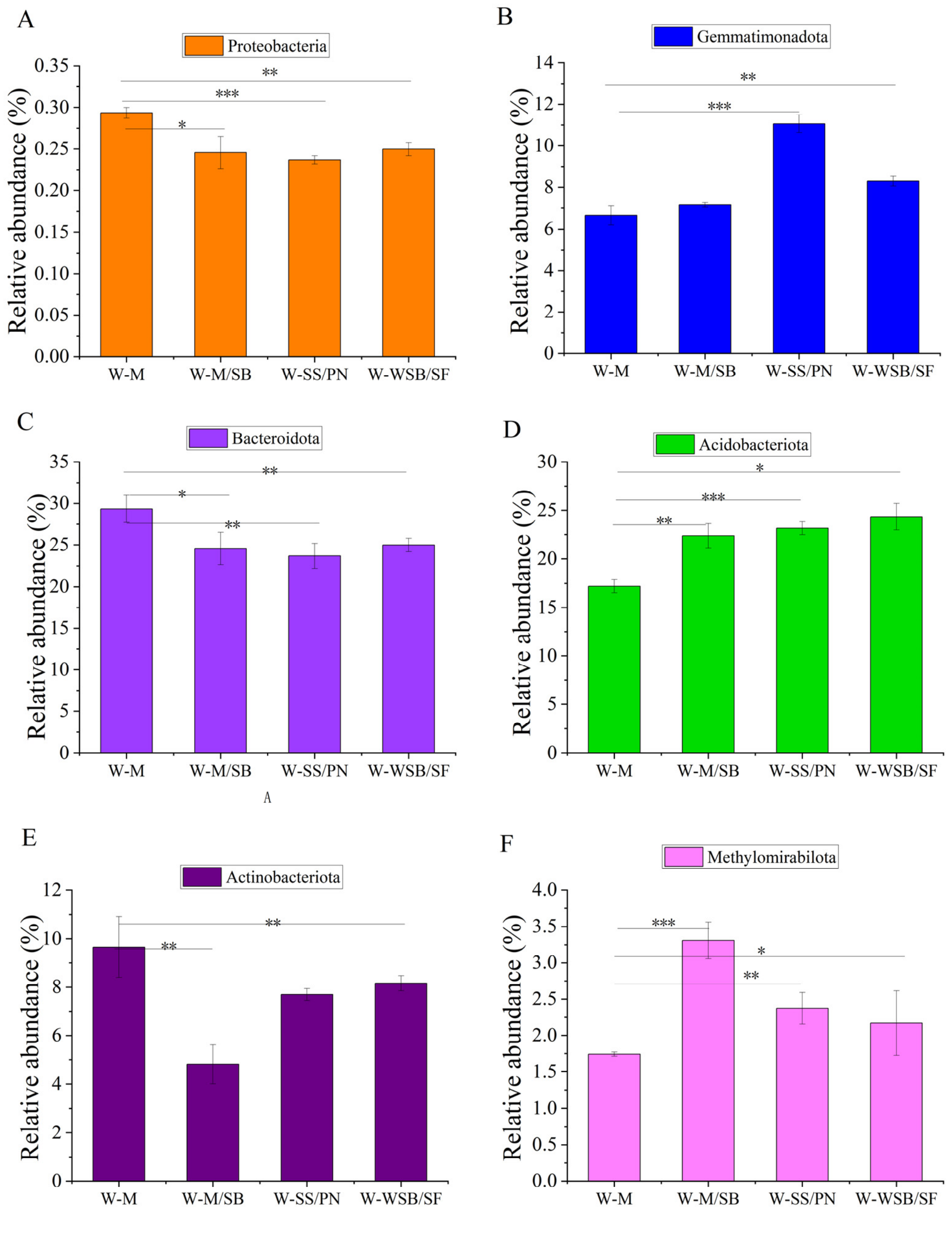

Figure S3 Species difference analysis of bacteria at the phylum level. (A) The relative abundance of Proteobacteria. (B) The relative abundance of Gemmatimonadota. (C) The relative abundance of Bacteroidota. (D) The relative abundance of Acidobacteriota. (E) The relative abundance of Actinobacteriota. (F) The relative abundance of Methylomirabilota. * $p < 0.05$, ** $p < 0.01$, *** $p < 0.001$.

Figure S4

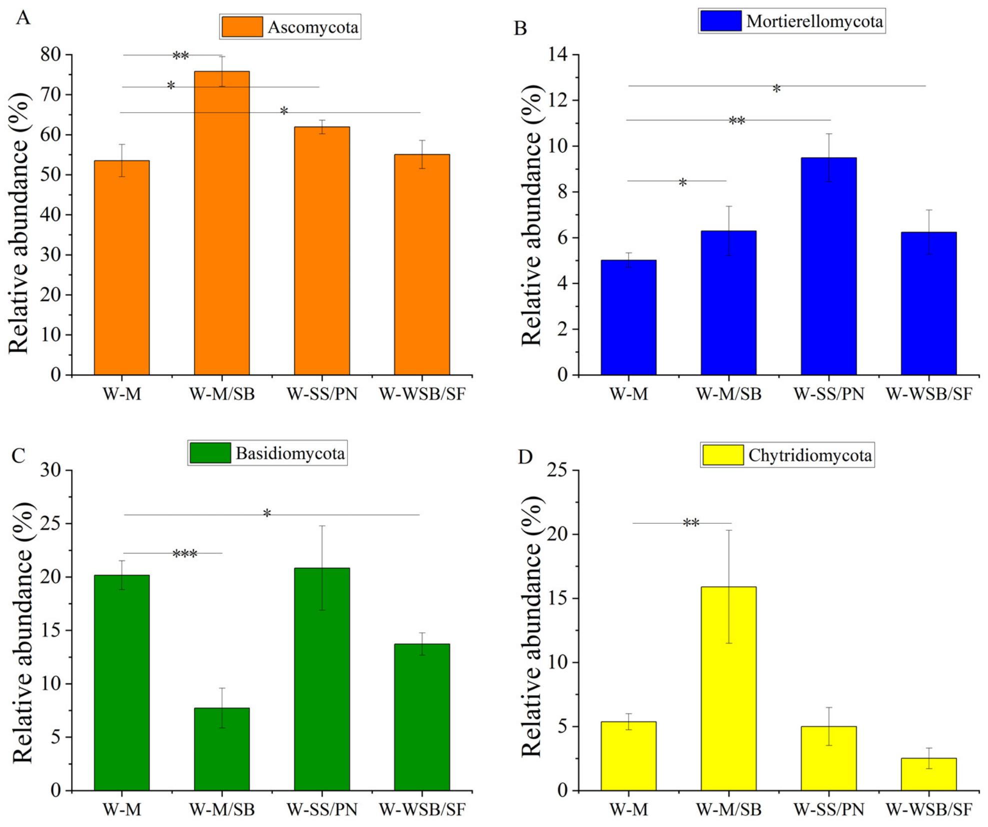

Figure S4 Species difference analysis of fungi at the phylum level. (A) The relative abundance of *Ascomycota*. (B) The relative abundance of *Mortierellomycota*. (C) The relative abundance of *Basidiomycota*. (D) The relative abundance of *Chytridiomycota*.* $p < 0.05$, ** $p < 0.01$, *** $p < 0.001$.
